# Editorial: Insights in synaptic neuroscience 2022

**DOI:** 10.3389/fnsyn.2024.1432259

**Published:** 2024-06-10

**Authors:** Karri P. Lamsa, Alfredo Kirkwood, P. Jesper Sjöström

**Affiliations:** ^1^Hungarian Centre of Excellence for Molecular Medicine Research Group for Human Neuron Physiology and Therapy, Szeged, Hungary; ^2^Department of Physiology, Anatomy and Neuroscience, University of Szeged, Szeged, Hungary; ^3^Department of Neuroscience, Mind/Brain Institute, Johns Hopkins University, Baltimore, MD, United States; ^4^Centre for Research in Neuroscience, Brain Repair and Integrative Neuroscience Program, Department of Neurology and Neurosurgery, The Research Institute of the McGill University Health Centre, Montreal General Hospital, Montreal, QC, Canada; ^5^Department of Medicine, The Research Institute of the McGill University Health Centre, Montreal General Hospital, Montreal, QC, Canada

**Keywords:** plasticity, synaptic release, neurotransmission, cortex, cell type

## Introduction

We are delighted to present the inaugural “*Insights in synaptic neuroscience 2022*” Research Topic. With this Research Topic, our goal was to shed light on the progress made in the past decade in the neuroscience field and on its future challenges to provide a thorough overview of the status of the art of the synaptic neuroscience field. We thus wished to showcase high-impact and authoritative articles on topical research at the frontiers of synaptic neuroscience, to highlight the diversity of research performed in the field of synaptic neuroscience. We originally aimed chiefly for review articles, but we are happy to report that the majority of works in this Research Topic ended up being original research. Below follows a brief editorial discourse of the papers in our Research Topic of articles.

## Papers in this Research Topic

### Long-term and structural forms of plasticity

In this Research Topic, Piette, Gervasi et al. reviews the recent development of synaptic plasticity investigation in intact brain in experimental animals. Fast development of methods and analyses of *in vivo* brain electrophysiology in unanesthetized animals, with molecular techniques to manipulate cellular plasticity mechanisms, enable neuronal plasticity studies under more and more naturalistic conditions in the brain. Piette, Gervasi et al. demonstrate with various examples how novel methodology allows us to study new research hypotheses closely linked to real-life brain functions and brain-body communication.

Chokshi et al. explored how the history of neural activity influences synaptic plasticity in the brain. Previous research showed that exposure to darkness followed by light re-exposure leads to a rapid weakening of excitatory synapses onto layer 2/3 pyramidal neurons in the primary visual cortex. The authors tested whether the period in the dark changed cortical plasticity such that the activity elicited by the re-exposure to light depressed the layer 2/3 synapses. To that end they first recorded *in vivo* in primary visual cortex the spike patterns evoked by re-exposure to light and subsequently they replayed theses spike patterns *ex-vivo* in slices from dark-exposed and in normal reared control mice. This stimulation, and also a Poisson pattern with the average rate both reduce the synaptic strength in the slices from dark-exposed missed, but not in the slices from control mice. These findings suggest that the history of visual experience alters how neurons in primary visual cortex respond to stimulation and that rapid synaptic depression can occur through various neural activity patterns.

Synaptic formation and establishment of long-term plasticity are orchestrated by protein complexes, and recent genomic and proteomic studies have uncovered new molecules possibly involved in these processes. In this Research Topic, León et al. provide an overview of the structure and molecular mechanisms by which glycoprotein M6a participates in synapse formation and maintenance. They summarize evidence collected from patients carrying mutations in the *GPM6A* gene, animal models, and *in vitro* studies that together suggest important role for M6a in normal function of synapses and in neurological conditions.

Tyro3 is a receptor tyrosine kinase that is expressed in neurons across several key brain areas such as striatum, neocortex, hypothalamus, and hippocampus. Despite this widespread expression, the role of Tyro3 has been unknown. Combining a battery of approaches—e.g., electron microscopy, patch-clamp electrophysiology, cell culturing, acute slices, and Tyro3 knockout mice—Miao et al. tightly link Tyro3 to the maturation of glutamatergic synapses. For instance, Tyro3 helps drive translocation of GluA2 AMPA receptor subunits to the plasma membrane. The authors propose that postsynaptic Tyro3 signals to coordinate activity that consolidates Hebbian synaptic plasticity. This study thus provides a hitherto unappreciated perspective on this receptor tyrosine kinase.

Another much more well-studied receptor is the ionotropic N-methyl-D-aspartate receptor (NMDAR), which is key mediator of glutamatergic neurotransmission and subcellular signaling. NMDARs famously signal via calcium to trigger synaptic plasticity, in particular long-term potentiation, but this capacity to flux calcium is to a large extent determined by NMDAR subunit composition. In their study, Beesley et al. reveal that NMDAR subunit composition can in fact be determined in acute brain slices using confocal microscopy and antibody labeling. This is an important achievement because elucidating NMDAR subunit composition is key to understanding NMDAR function, yet the subunit-specific pharmacology for NMDARs has long been less than adequate.

Endogenous release of nitric oxide plays a key role in the induction and establishment of synaptic long-term potentiation of excitatory synapses to hippocampal pyramidal cell apical dendrites, but not to their basal dendrites. Ivanova et al. compared synaptic inputs to apical and basal dendrites of these neurons and found a difference in their function indicating distinct contribution of GluA2-lacking AMPA receptors. Although the GluA2-lacking receptors are present both in apical and basal dendrites, their contribution to synaptic transmission in apical dendrites is higher than in basal dendrites, and the nitric oxide synthase blockade flattens this difference. The results show that inhibition of nitric oxide synthase selectively reduces signaling through GluA2-lacking AMPAR to the apical dendrites, a feature which may contribute to establishment long-term potentiation in these excitatory synapses.

Endocannabinoids are important signaling molecules during neuronal development in the brain and retina, and they regulate maturation of axons of projection neurons. Del Rio et al. analyzed axonal arbors of retinal ganglion cells following targeted downregulation of cannabinoid receptor type 1 (CB1R) in Xenopus tadpoles and exposing them to pharmacologic agents. They found that retinal ganglion cells modulated their axonal growth through CB1R signaling, and that the endogenous cannabinoid signaling also contributed to structural differentiation of postsynaptic neuron dendrites in optic tectum in the brain. Their study shows that alterations in endocannabinoid levels in a developing embryo regulated neuronal connectivity through pre- and postsynaptic sites in the intact brain. The study highlights sensitivity of developing brain to exposure to cannabinoids.

### Synaptic release and neurotransmission

In neurotransmitter release, four modes of synaptic vesicle cycling have been identified, yet it has remained unclear which are most biologically relevant. To address this, Paksoy et al. explored the role of clathrin in synaptic vesicle recycling at the calyx of Held under physiological conditions. They relied on the clathrin inhibitor Pitstop-2 combined with serial sectioning scanning electron microscopy to reveal that, under biologically plausible conditions, clathrin plays a role in synaptic vesicle recycling from both large endosomes as well as the plasma membrane.

The balance between glutamatergic excitatory and GABAergic inhibitory synaptic transmission is crucial for information processing in the neocortex. Moreover, even transient disruptions in this balance during early development can contribute to neuropsychiatric disorders later in life. Notably, in the KI mouse line mice the haplodeficiency of the GABA synthetizing enzyme GAD67 does not result in epileptic activity and only in mild behavioral deficits. The examination by Ueberbach et al. confirmed the expected decrease in fast inhibitory synaptic activity in in this mouse line, yet network activity was seemingly balanced by a compensatory increased tonic activation of the slow GABA_B_ receptors. In turn, the enhanced GABA_B_ receptors were likely to result from increased GABA release via GABA transporter 3 (GAT-3) working in reverse mode.

### Cell types of deep cortical layers

The frontal eye field in primates contains diverse neurons with different functions. While some neurons signal movement and fixation, others also respond to visual stimuli. However, identifying distinct cell types has been challenging. In this Research Topic, Piette, Vandecasteele et al. examined biophysical properties of deep layer pyramidal neurons in slices and identified two main subtypes: one with low resistance and low excitability, and another with high resistance and strong excitability. These findings suggest the presence of at least two distinct populations of deep-layer neurons in the frontal eye field, which has implications for understanding visual attention and saccade production.

The precuneus—which is part of the human posteromedial cortex—contributes to processing of multimodal information including emotion, social behavior, and spatial cognition. In an anatomical study of human brain tissue that relied on Nissl and Golgi stains, Fuentealba-Villarroel et al. reveal that the presence of elongated spindle-shaped neurons in cortical layer 5 that are suggestive of von Economo neurons. These neurons are of interest, because they can be found in humans and other primates, but not in all mammals. A tentative link between these neurons and neuropsychiatric disorders involving the precuneus highlights the need for a better and more detailed understanding of this intriguing cell type.

## Concluding remarks

To summarize, this Research Topic has highlighted recent advances in synaptic neuroscience. We hope and expect that this Research Topic will inspire, inform, and provide direction and guidance to researchers in the emerging subfield of synaptic neuroscience. We would like to extend our gratitude to all the authors who contributed.

## Author contributions

KL: Writing – original draft, Writing – review & editing. AK: Writing – original draft, Writing – review & editing. PJS: Writing – original draft, Writing – review & editing.

